# Supplementary material for: Complementary use of autoantibody detection methods facilitates diagnosis of juvenile autoimmune hepatitis and autoimmune sclerosing cholangitis
Source: JHEP Rep. 2025 Dec 6;8(2):101706. doi: 10.1016/j.jhepr.2025.101706 (PMC12857373; doi:10.1016/j.jhepr.2025.101706)
Supplement: Multimedia component 1 [file mmc1.pdf]

Complementary use of autoantibody detection methods facilitates diagnosis of juvenile autoimmune hepatitis and autoimmune sclerosing cholangitis

Theresa Kirchner, Norman Junge, Nicole Henjes, Stephanie Loges, Muhammed Yuksel, Wojciech Janczyk, Claudine Lalanne, Kalliopi Zachou, Ye H. Oo, Jérôme Gournay, Simon Pape, Joost P.H. Drenth, Amédée Renand, George N. Dalekos, Luigi Muratori, Piotr Socha, Cigdem Arikan, Yun Ma, Heiner Wedemeyer, Ulrich Baumann, Bastian Engel, Richard Taubert

Table of contents

Fig. S1……………………………………………………………………………….…2

Fig. S2………………………………………………………………………………..…3

Fig. S3…………………………………………………………………………………4

Table S1……………………………………………………………….………...….5

Table S2………………………………………………………………………….…....5

Table S3……………………………………………………………………….……....6

Table S4……………………………………………………………………………….....7

Table S5…………………………………………………………………….……….....7

Table S6…………………………………………………………………….……….....8

Table S7…………………………………………………………………….……….....9

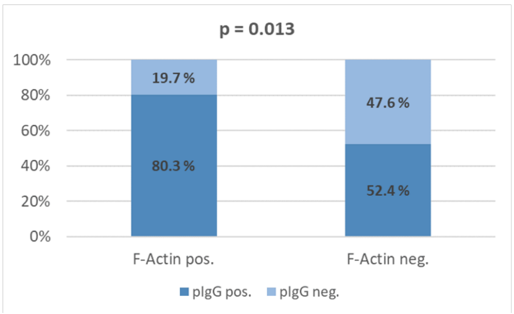

Fig. S1: Frequency of pIgG positive samples dependent on presence of anti-F-actin antibodies

Stacked graphs show frequencies of pIgG positive samples (dark blue) and negative samples (light blue) in children with AIH/AISC with positivity (F-Actin pos.) or negativity (F-Actin neg.) for F-Actin. Chi² test was used for statistical comparison.

A

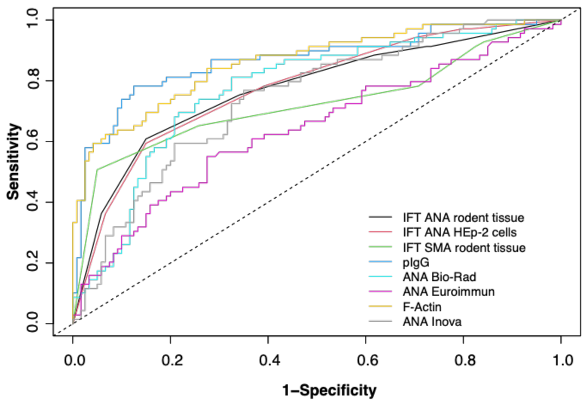

B

		AUC	95% CI	
			LowerLimit	UpperLimit
ELISA	F-Actin ELISA	.861	.804	.917
	PIgG	.866	.807	.925
	ANA Bio-Rad	.774	.704	.843
	ANA Inova	.740	.669	.812
	ANA Euroimmun	.640	.555	.724
IFT	IFT ANA rodent tissue	.771	.699	.843
	IFT SMA	.723	.640	.806
	IFT ANA Hep-2	.777	.709	.806

C

<table>
<tr><th></th><th></th><th>adjusted p</th></tr>
<tr><td rowspan="7">IFT ANA rodent tissue</td><td>IFT SMA</td><td rowspan="5">1.000</td></tr>
<tr><td>IFT ANA HEp-2 cells</td></tr>
<tr><td>pIgG</td></tr>
<tr><td>F-Actin</td></tr>
<tr><td>ANA Inova</td></tr>
<tr><td>ANA Euroimmun</td><td>0.576</td></tr>
<tr><td>ANA Bio-Rad</td><td>1.000</td></tr>
<tr><td rowspan="6">IFT SMA</td><td>IFT ANA HEp-2 cells</td><td>1.000</td></tr>
<tr><td>pIgG</td><td>0.163</td></tr>
<tr><td>F-Actin</td><td>0.197</td></tr>
<tr><td>ANA Inova</td><td rowspan="3">1.000</td></tr>
<tr><td>ANA Euroimmun</td></tr>
<tr><td>ANA Bio-Rad</td></tr>
<tr><td rowspan="5">IFT ANA HEp-2 cells</td><td>pIgG</td><td rowspan="3">1.000</td></tr>
<tr><td>F-Actin</td></tr>
<tr><td>ANA Inova</td></tr>
<tr><td>ANA Euroimmun</td><td>0.376</td></tr>
<tr><td>ANA Bio-Rad</td><td>1.000</td></tr>
<tr><td rowspan="4">pIgG</td><td>F-Actin</td><td>1.000</td></tr>
<tr><td>ANA Inova</td><td>0.226</td></tr>
<tr><td>ANA Euroimmun</td><td>< 0.001</td></tr>
<tr><td>ANA Bio-Rad</td><td>1.000</td></tr>
<tr><td rowspan="3">F-Actin</td><td>ANA Inova</td><td>0.275</td></tr>
<tr><td>ANA Euroimmun</td><td>< 0.001</td></tr>
<tr><td>ANA Bio-Rad</td><td>1.000</td></tr>
<tr><td rowspan="2">ANA Inova</td><td>ANA Euroimmun</td><td rowspan="2">1.000</td></tr>
<tr><td>ANA Bio-Rad</td></tr>
<tr><td>ANA Euroimmun</td><td>ANA Bio-Rad</td><td>0.467</td></tr>
</table>

Fig. S2: Test performance of different assays for the diagnosis of treatment-naive juvenile AIH

(A+B) ROC curves including the corresponding AUCs and 95% confidence intervals for the diagnosis of AIH by different assays. (C) DeLong test with adjustment for multiple comparisons with the Bonferroni method indicating the p-value for the respective comparisons of AUCs between assays. IFT: immunofluorescence testing; ANA: antinuclear antibodies; SMA: anti-smooth muscle antibodies; pIgG: polyreactive immunoglobulin G; HEp-2: human epithelioma-2

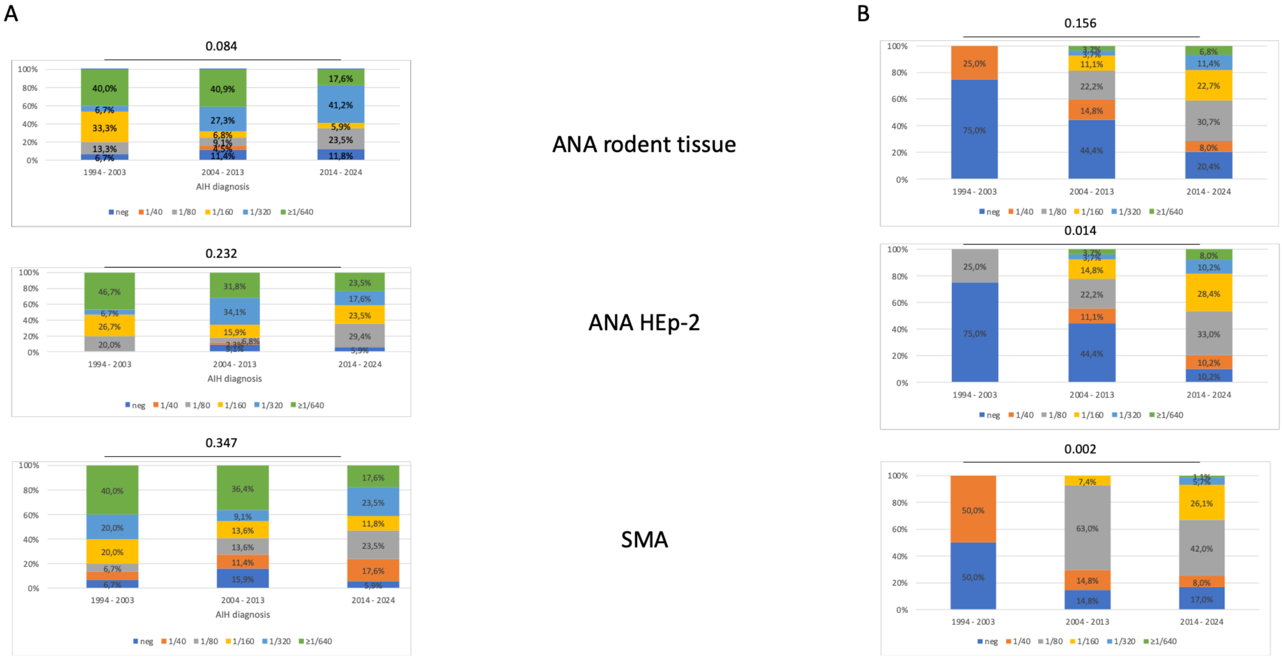

Fig. S3: Frequency of antibody titres in AIH/AISC and controls

Stacked graphs show frequencies of different ANA titres on rodent tissue, HEp-2 cells and SMA titres on rodent tissue in AIH/AISC (A) and non-AIH-LD (B) across three different decades of sample collection. Chi² test was used for statistical comparison.

Table S1: Frequency of non-AIH-non-AISC liver disease controls

n=120	n (%)
MASLD	37 (30.8)
PFIC	8 (6.7)
PSC	13 (10.8)
toxic	4 (3.3)
Wilson's disease	35 (29.22)
alpha 1 antitrypsin deficiency	7 (5.8)
unknown	9 (7.5)
other	7 (5.8)

MASLD: metabolic-dysfunction associated steatotic liver disease; PFIC: progressive familiary intrahepatic cholestasis; PSC: primary sclerosing cholangitis. Other: cytochrome oxidase deficiency, congenital hepatitis with portal hypertension, acute liver failure of unknown aetiology, primary hyperoxaluria, trisomie 21, celiac disease, giant cell hepatitis

Table S2: Staining patterns of ANA on HEp-2 cells

		AIH/AISC	Non-AIH-LD	p
		n (%)	n (%)	
ANA Pattern	fine speckled	30 (39.0 %)	69 (70.4 %)	.000
	homogenous	39 (50.7 %)	22 (22.5 %)	.001
	fine speckled/homogenous	6 (7.8 %)	3 (3.1 %)	.165
	nucleolar	1 (1.3 %)	4 (4.1 %)	.272
	centromer	1 (1.3 %)	0 (0.0 %)	.259

ANA: antinuclear antibodies; AIH: autoimmune hepatitis; AISC: autoimmune sclerosing cholangitis; non-AIH-LD: non-AIH, non-autoimmune sclerosing cholangitis liver disease. Mann-Whitney U test.

Table S3: Test performance of IFT-based autoantibody detection in treatment-naive juvenile AIH vs. non-AIH-LD

ANA IFT	Titer	sensitivity	specificity	PPV	NPV	accuracy
Any tissue positivity	1:20	89,9%	26,7%	41,3%	82,1%	49,7%
	1:40	89,9%	27,5%	41,6%	82,5%	50,3%
	1:80	87,0%	38,3%	44,8%	83,6%	56,1%
	1:160	73,9%	65,8%	55,4%	81,4%	68,8%
	**1:320**	**59,4%**	**85,0%**	**69,5%**	**78,5%**	**75,7%**
	1:640	34,8%	94,2%	77,4%	71,5%	72,5%
HEp-2 cell positivity	1:20	95,7%	18,3%	40,2%	88,0%	46,6%
	1:40	95,7%	20,0%	40,7%	88,9%	47,6%
	1:80	92,8%	30,0%	43,2%	87,8%	52,9%
	1:160	76,8%	60,8%	53,0%	82,0%	66,7%
	**1:320**	**58,0%**	**85,0%**	**69,0%**	**77,9%**	**75,1%**
	1:640	34,8%	93,3%	75,0%	71,3%	72,0%
**SMA IFT**						
any SMA	1:20	92,8%	15,8%	38,8%	79,2%	43,9%
	1:40	91,3%	18,5%	38,9%	77,8%	44,4%
	1:80	78,3%	29,2%	38,9%	70,0%	47,1%
	1:160	65,2%	74,2%	59,2%	78,8%	70,9%
	**1:320**	**50,7%**	**95,0%**	**85,4%**	**77,0%**	**78,8%**
	1:640	34,8%	99,2%	96,0%	72,6%	75,7%
V	1:20	11,6%	75,0%	21,1%	59,6%	51,9%
	1:40	11,6%	75,0%	21,1%	59,6%	51,9%
	1:80	8,7%	79,2%	19,4%	60,1%	53,4%
	1:160	4,4%	92,5%	25,0%	62,7%	60,3%
	**1:320**	**1,5%**	**98,3%**	**33,3%**	**63,4%**	**63,0%**
	1:640	0,0%	1000,0%		63,5%	63,5%
VG	1:20	50,7%	70,0%	49,3%	71,2%	63,0%
	1:40	49,3%	71,7%	50,0%	71,1%	63,5%
	1:80	44,9%	76,7%	52,5%	70,8%	65,1%
	1:160	43,5%	93,3%	79,0%	74,2%	75,1%
	**1:320**	**37,7%**	**99,2%**	**96,3%**	**73,5%**	**76,7%**
	1:640	27,5%	100,0%	100,0%	70,6%	73,5%
VGT	**1:20**	**13,00%**	**100,00%**	**100,00%**	**66,70%**	**68,30%**
	1:40	13,00%	100,00%	100,00%	66,70%	68,30%
	1:80	13,00%	100,00%	100,00%	66,70%	68,30%
	1:160	13,00%	100,00%	100,00%	66,70%	68,30%
	1:320	11,60%	100,00%	100,00%	66,30%	67,70%
	1:640	11,60%	100,00%	100,00%	66,30%	67,70%

ANA: antinuclear antibodies. SMA: anti-smooth muscle antibodies. PPV: positive predictive value. NPV: negative predictive value. V: staining of vessels. VG: staining of vessels and glomeruli. VGT: staining of vessels, glomeruli and tubuli. IFT: immunofluorescence testing.

Table S4: Test performance of ELISA-based autoantibody in treatment-naive juvenile AIH vs. non-AIH-LD

recommended cut offs	ANA				F-Actin		pIgG
	Euroimmun	Bio-Rad	Inova				
Cut-off	≥1.0	≥1.0	>20(-60)	>60	>20 (-30)	>30	1.27 nAU
Sensitivity	24,6%	60,9%	62,3%	20,3%	73,9%	60,9%	78,3%
Specificity	90,8%	80,0%	69,2%	94,2%	80,0%	94,2%	86,7%
PPV	60,7%	63,6%	53,8%	66,7%	68,0%	85,7%	77,1%
NPV	67,7%	78,1%	76,2%	67,3%	84,2%	80,7%	87,4%
Accuracy	66,7%	73,0%	66,7%	67,2%	77,8%	82,0%	83,6%

ANA: antinuclear antibodies. PPV: positive predictive value. NPV: negative predictive value. pIgG: polyreactive immunoglobulin G.

Table S5: Agreement between different assays

		kappa
IFT ANA rodent tissue ≥1/20	IFT ANA HEp-2 ≥1/20	0.71
	pIgG	0.04
	ANA Inova	0.12
	ANA Bio-Rad	0.12
	ANA Euroimmun	0.03
IFT ANA rodent tissue ≥1/320	IFT ANA HEp-2 ≥1/320	0.81
	pIgG	0.32
	ANA Inova	0.31
	ANA Bio-Rad	0.40
	ANA Euroimmun	0.19
IFT ANA HEp-2 ≥1/20	pIgG	0.01
	ANA Inova	0.08
	ANA Bio-Rad	0.07
	ANA Euroimmun	0.02
IFT ANA HEp-2 ≥1/320	pIgG	0.30
	ANA Inova	0.36
	ANA Bio-Rad	0.41
	ANA Euroimmun	0.20
IFT any SMA ≥1/20	F-Actin	0.02
IFT any SMA ≥1/320	F-Actin	0.39
pIgG	F-Actin	0.45

Agreement is assessed by Cohen's Kappa ("Kappa"). Titer is provided as cut-off value for IFT-based assays.

pIgG: polyreactive immunoglobulin G; IFT: immunofluorescence testing; ANA: antinuclear antibodies; SMA: anti-smooth muscle antibodies: HEp-2: human epithelioma 2.

Table S6: Sensitivity and Specificity of ELISA with regard to storage duration

pIgG: polyreactive immunoglobulin G; ANA: antinuclear antibodies. Chi² test.

Table S7: Correlation of IgG with different autoantibody tests

Pearson correlation coefficient is provided. ANA: antinuclear antibodies. SMA: anti-smooth muscle antibodies: HEp-2: human epithelioma 2. pIgG: polyreactive immunoglobulin G. IgG: immunoglobulin G.
