# Supplementary material for: Complementary use of autoantibody detection methods facilitates diagnosis of juvenile autoimmune hepatitis and autoimmune sclerosing cholangitis
Source: JHEP Rep. 2025 Dec 6;8(2):101706. doi: 10.1016/j.jhepr.2025.101706 (PMC12857373; doi:10.1016/j.jhepr.2025.101706)
Supplement: Multimedia component 2 [file mmc2.docx]

**JHEP Reports**

**CTAT methods**

Tables for a “Complete, Transparent, Accurate and Timely account” (CTAT) are now mandatory for all revised submissions. The aim is to enhance the reproducibility of methods.

- Only include the parts relevant to your study
- Refer to the CTAT in the main text as ‘Supplementary CTAT Table’
- Do not add subheadings
- Add as many rows as needed to include all information
- Only include one item per row

**If the CTAT form is not relevant to your study, please outline the reasons why:**

|  |
| --- |

- 1. **Antibodies**

| **Name** | **Citation** | **Supplier** | **Cat no.** | **Clone no.** |
| --- | --- | --- | --- | --- |
| **Peroxidase-conjugated AffiniPure Rabbit Anti-Human IgG (H+L)** |  | **Jackson ImmunoResearch Europe Ltd.** | **309-035-003** | **polyclonal** |

- 1. **Cell lines**

| **Name** | **Citation** | **Supplier** | **Cat no.** | **Passage no.** | **Authentication test method** |
| --- | --- | --- | --- | --- | --- |
|  |  |  |  |  |  |

- 1. **Organisms**

| **Name** | **Citation** | **Supplier** | **Strain** | **Sex** | **Age** | **Overall n number** |
| --- | --- | --- | --- | --- | --- | --- |
|  |  |  |  |  |  |  |

- 1. **Sequence based reagents**

| **Name** | **Sequence** | **Supplier** |
| --- | --- | --- |
|  |  |  |

- 1. **Biological samples**

| **Description** | **Source** | **Identifier** |
| --- | --- | --- |
| **Human serum samples** | **Local biorepositories** | **n/a** |

- 1. **Deposited data**

| **Name of repository** | **Identifier** | **Link** |
| --- | --- | --- |
|  |  |  |

- 1. **Software**

| **Software name** | **Manufacturer** | **Version** |
| --- | --- | --- |
| **SPSS** | **SPSS** | **22** |
| **R Statistical Software** | **R Core Team** | **4.1.2** |
| **GraphPad Prism** | **GraphPad Software** | **6** |

- 1. **Other (*e.g*. drugs, proteins, vectors etc.)**

| **Quanta Lite Actin IgG ELISA** | Inova Diagnostics, USA | **Order number 708785** |
| --- | --- | --- |
| **Quanta Lite ANA ELISA** | **Inova Diagnostics, USA** | order number 708750 |
| ANA Screening Test | Bio-Rad Laboratories, Inc., Hercules, CA, USA | order number 96AN |
| ANA Screen ELISA | Euroimmun Medizinische Labordiagnostika AG, Lübeck, Germany | order number EA 1590-9601-11 G |
| AESKUSLIDES®, | AESKU-Diagnostics, Wendelsheim, Germany | Order No.: 517.050.Bulk5 |
| NOVA Lite® HEp-2 ANA Kits/Substrate Slides | Inova Diagnostics, USA | Order No: 508100.20 |
| **HIP1R** | **MGQLQDQQALRHMQASLVRTPLQGILQLGQELKPKSLDVRQE** | **Biomatik, Cambridge, Ontario/Canada** |
| **3, 3', 5, 5'**  **tetramethyl benzidine** |  | **BioLegend, San Diego California** |
| **Olympus BX60 microscope** | **Evident Europe GmbH, Germany** |  |

- 1. **Please provide the details of the corresponding methods author for the manuscript:**

| **Dr. Bastian Engel**  **Hannover Medical School**  **Department of Gastroenterology, Hepatology, Infectious Diseases and Endocrinology**  **Carl-Neuberg-Straße 1**  **30625 Hannover**  **Germany**  **Engel.Bastian@mh-hannover.de** |
| --- |

**2.0 Please confirm for randomised controlled trials all versions of the clinical protocol are included in the submission. These will be published online as supplementary information.**

|  |
| --- |
