# Supplementary material for: Complementary use of autoantibody detection methods facilitates diagnosis of juvenile autoimmune hepatitis and autoimmune sclerosing cholangitis
Source: JHEP Rep. 2025 Dec 6;8(2):101706. doi: 10.1016/j.jhepr.2025.101706 (PMC12857373; doi:10.1016/j.jhepr.2025.101706)
Supplement: Multimedia component 4 [file mmc4.pdf]

Research article
Paediatric hepatology

JHEP|Reports

Complementary use of autoantibody detection methods facilitates diagnosis of juvenile autoimmune hepatitis and autoimmune sclerosing cholangitis

Authors

Theresa Kirchner, Norman Junge, Nicole Henjes, …, Ulrich Baumann, Bastian Engel, **Richard Taubert,**

Correspondence

Engel.Bastian@mh-hannover.de (B. Engel), taubert.richard@mh-hannover.de (R. Taubert).

Graphical abstract

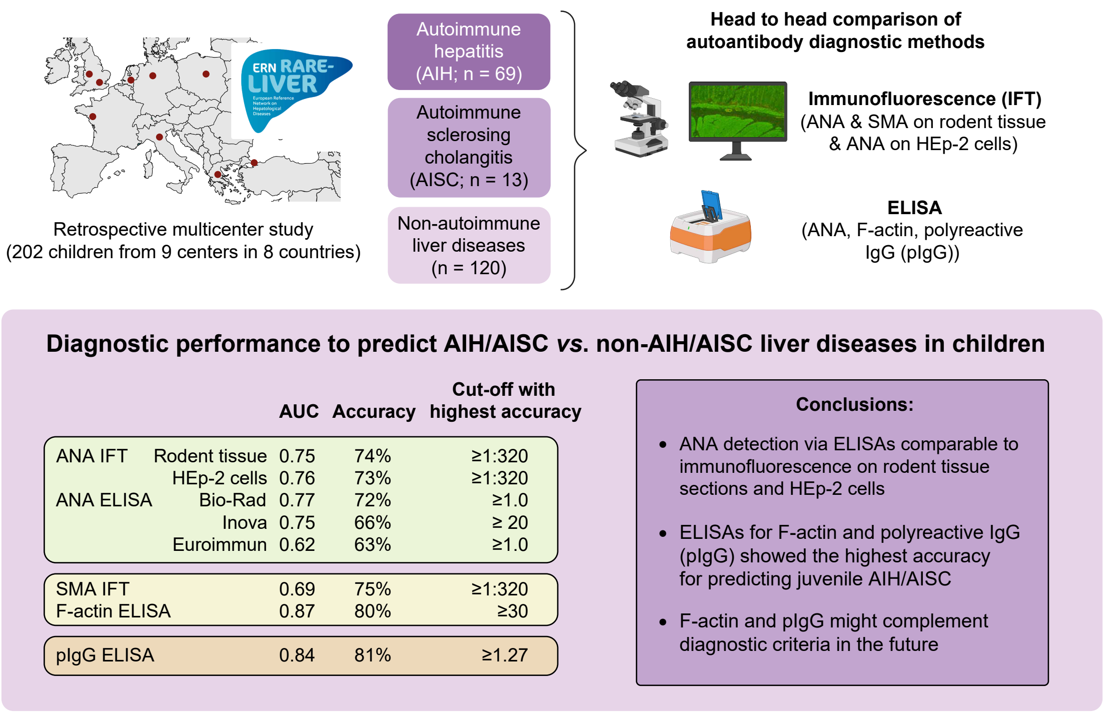

Highlights:

- Optimal IFT accuracy for ANA and SMA occurred at a 1:320 dilution.
- SMA VGT staining pattern achieved 100% specificity.
- ANA ELISAs and IFT on HEp-2 cells performed comparably to IFT on rodent tissue.
- F-actin and pIgG ELISAs showed the highest AUCs for juvenile AIH/AISC.
- pIgG detected 52% of F-actin-negative AIH, increasing diagnostic yield.

Impact and implications:

Autoimmune hepatitis (AIH) and autoimmune sclerosing cholangitis (AISC) are rare paediatric liver diseases that can be difficult to distinguish from other hepatopathies. Autoantibody testing is central to diagnosis, yet paediatric performance across platforms has been poorly standardised and sparsely reported. In this multicentre comparison (202 sera from nine expert centres across eight European countries), indirect immunofluorescence (IFT) on rodent tissue showed suboptimal discrimination at the commonly advocated 1:20 cut-off, whereas accuracy for ANA and SMA improved markedly at 1:320. ANA detection by IFT on HEp-2 cells and by ELISA was comparable to rodent tissue IFT. ELISAs for F-actin and pIgG achieved the highest AUCs for identifying AIH/AISC and may complement current diagnostics. Substantial inter-platform discordance for ANA underscores the need for harmonisation. Collectively, these data support the use of multiple validated platforms with platform-appropriate cut-offs in paediatric AIH/AISC serology and support updates to diagnostic algorithms to improve diagnostic timeliness and reliability.

https://doi.org/10.1016/j.jhepr.2025.101706
© 2025 The Author(s). Published by Elsevier B.V. on behalf of European Association for the Study of the Liver (EASL). This is an open access article under the CC BY license (http://creativecommons.org/licenses/by/4.0/). JHEP Reports, 2026, 8, 1–10

JHEP|Reports

Complementary use of autoantibody detection methods facilitates diagnosis of juvenile autoimmune hepatitis and autoimmune sclerosing cholangitis

Theresa Kirchner[1,14], Norman Junge[2,14], Nicole Henjes[1,14], Stephanie Loges[1,14], Muhammed Yuksel[3,4], Wojciech Janczyk[5,14], Claudine Lalanne[6,14], Kalliopi Zachou[7,8,14], Ye H. Oo[9,14], Jérôme Gournay[10], Simon Pape[11,14], Joost PH. Drenth[12,14], Amédée Renand[13], George N. Dalekos[7,8,14], Luigi Muratori[6,14], Piotr Socha[5,14], Cigdem Arikan[4], Yun Ma[3], Heiner Wedemeyer[1,14], Ulrich Baumann[2,14], **Bastian Engel**[1,14,15,*,†], **Richard Taubert**[1,14,*,†]

JHEP Reports **2026**. vol. 8 | 1–10

Check for updates

Background & Aims: The diagnosis of juvenile autoimmune hepatitis (AIH) is challenging given its heterogeneous presentation. Autoantibodies, typically detected by immunofluorescence testing (IFT), together with liver histology, represent key diagnostic features. Polyreactive immunoglobulin G (pIgG) has recently emerged as a complementary biomarker in AIH. This retrospective multicentre study aimed to compare ELISA-based autoantibody testing and IFT on HEp-2 cells with the gold standard of IFT on rodent tissue sections in children with autoimmune and non-autoimmune liver diseases.

Methods: Autoantibody detection was performed centrally at Hannover Medical School using three commercial antinuclear antibody (ANA) ELISAs, one commercial F-actin ELISA, one in-house pIgG ELISA, and IFT on HEp-2 cells, in comparison to the gold standard of IFT on rodent tissue sections. Samples from children with AIH (n = 69), autoimmune sclerosing cholangitis (AISC; n = 13) and other liver diseases (n = 120) were analysed from nine European centres.

Results: The AUCs for the detection of AIH/AISC were moderate to good for ANA detection by IFT (gold standard of rodent tissue AUC: 0.748; HEp-2 AUC: 0.756) and were comparable to ELISA-based detection (0.622-0.772). Anti-smooth muscle antibody (SMA) IFT on rodent tissue yielded an AUC of 0.694. Specificity was increased to 100% by including the SMA staining pattern of vessels, glomeruli and tubules. ELISA-based quantification of anti-F-actin (AUC = 0.868) and pIgG (AUC = 0.844) showed the highest AUCs. While the majority of F-actin–positive children were pIgG-positive (80.3%), pIgG was also detected in 52.4% of F-actin–negative children with AIH.

Conclusion: ELISA-based assays provide reliable ANA detection comparable to IFT. Anti-F-actin and pIgG ELISAs showed the highest accuracy for predicting juvenile AIH/AISC and may complement existing diagnostic criteria.

© 2025 The Author(s). Published by Elsevier B.V. on behalf of European Association for the Study of the Liver (EASL). This is an open access article under the CC BY license (http://creativecommons.org/licenses/by/4.0/).

Introduction

Juvenile autoimmune hepatitis (AIH) is a rare disease affecting girls more often than boys.[1] It is characterised by circulating autoantibodies, polyclonal hypergammaglobulinemia with elevated immunoglobulin G (IgG), lymphoplasmacytic inflammatory lesions and interface hepatitis on liver histology, and the exclusion of competing aetiologies of liver disease.[1] Diagnosis remains challenging as there is considerable overlap with other liver diseases, such as metabolic dysfunction-associated steatotic liver disease, drug-induced liver injury or Wilson's disease.[2,3] Juvenile AIH can be associated with biliary autoimmune manifestations, such as autoimmune sclerosing cholangitis (AISC). The diagnosis of AIH/AISC is mostly suspected by elevated autoantibody titres in the blood.

The International Autoimmune Hepatitis Group (IAIHG) recommends the assessment of antinuclear antibodies (ANA), anti-smooth muscle antibodies (SMA), anti-liver kidney microsomal type 1 antibodies and anti-liver cytosol antibodies with immunofluorescence testing (IFT) on rodent tissue sections.[4] The scoring system suggests specific cut-off values for the interpretation of IFT in children (at least 1:20), which are significantly lower than in adults (at least 1:40).[1] Recently, in adults, ANA scoring on human epithelioma-2 (HEp-2) cells was shown to be as accurate as scoring on rodent tissue sections when different cut-offs were applied and when scoring was supplemented by anti-F-actin antibody positivity, which was not part of the original scoring system.[5–8] While ELISA has the potential to reduce interobserver variability and is less time-consuming than IFT, recent data in adults suggest that it

* Corresponding authors. Address: Hannover Medical School, Department of Gastroenterology, Hepatology, Infectious Diseases and Endocrinology, Carl-Neuberg-Straße 1, 30625 Hannover, Germany.
E-mail addresses: Engel.Bastian@mh-hannover.de (B. Engel), taubert.richard@mh-hannover.de (R. Taubert).
† Richard Taubert and Bastian Engel were equally contributed.
https://doi.org/10.1016/j.jhepr.2025.101706

may replace IFT for ANA detection.[5] In terms of new antibody candidates for the diagnosis of AIH, the quantification of polyreactive IgG (pIgG) has recently been proposed to complement current diagnostics.[9,10] The unmet need for standardisation of autoantibody detection between different test systems and centres has recently been highlighted by the IAIHG.[11] IFT on rodent tissue sections, endorsed by the IAIHG[4] as the gold standard for the detection of ANA and SMA, is highly operator dependent, lacks inter-centre standardisation and is more time-consuming than ELISA, the latter being more commonly used in the USA.[5,12] Findings in adults may not be readily extrapolated to children and, to our knowledge, there are no comparative studies reporting head-to-head comparisons of IFT-based and ELISA-based testing for autoantibodies in paediatric AIH/AISC.

The objectives of the study were to evaluate the diagnostic value of ELISA-based autoantibody detection and IFT on HEp-2 cells compared to the recommended gold standard of IFT on rodent tissue sections for which we recruited an independent retrospective multicentre cohort that resembles the clinical scenario of identifying AIH/AISC from a variety of non-viral hepatopathies.

Patients and methods

Study design and population

This retrospective multicentre study included 202 children (juvenile AIH n = 69, AISC n = 13, non-AIH, non-AISC liver disease [non-AIH-LD, n = 120]) from nine European expert centres in eight countries (Fig. 1). The diagnosis of AIH and AISC was based on the published scoring system[1] and subsequently confirmed by disease behaviour at follow-up by the respective treating physicians. For simplicity, non-AIH and non-AISC liver diseases will be summarized as non-AIH-LD. Non-AIH-LD was diagnosed according to current guidelines and, for alpha-1 antitrypsin deficiency, by reduced serum alpha-1 antitrypsin and genotyping.[13–16] All samples were collected at the time of diagnosis from children who were not receiving immunosuppressive therapy at the time of sampling and had not received such treatment prior to sampling. Children with informed consent and available biomaterial were considered eligible for inclusion by the local investigators. Samples were collected between 1994 and 2020 and stored

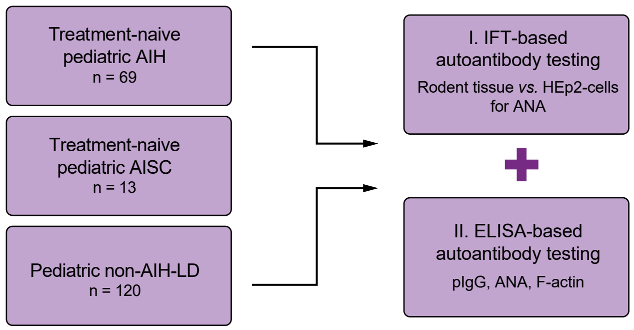

Fig. 1. Study design. AIH, autoimmune hepatitis; AISC, autoimmune sclerosing cholangitis; ANA, antinuclear antibodies; HEp-2, human epithelioma-2; IFT, immunofluorescence testing; non-AIH-LD, non-AIH, non-AISC liver disease; pIgG, polyreactive immunoglobulin G; SMA, anti-smooth muscle antibodies.

frozen at -80 °C until use in this study, for which they were identified retrospectively from existing biorepositories. The study was approved by the Ethics Committee of Hannover Medical School (MHH Ethikkommission, Hannover, Germany; approval numbers 1025-2011 and 2665-2015). Written informed consent was obtained from parents and children at all centres, as appropriate. The use of retained samples from the clinical laboratories of Hannover Medical School, collected from paediatric patients with liver disease, was approved by the local ethics committee (approval number 2817-2015). The use of material and data from children from the other centres was approved by the respective local ethics committees. The study adhered to the ethical guidelines of the 1975 Declaration of Helsinki. All experiments were performed in accordance with the relevant guidelines and regulations.

Autoantibody testing by IFT

All IFTs were performed at Hannover Medical School, Germany, by experienced technicians blinded to the diagnosis and to any pre-existing external test results and clinical data. The presence of autoantibodies (ANA and SMA) in all serum samples was manually tested by IFT on sections of rodent liver, stomach and kidney using commercially available substrates (AESKUSLIDES®, AESKU-Diagnostics, Wendelsheim, Germany. Order No.: 517.050.Bulk5; NOVA Lite® HEp-2 ANA Kits/ Substrate Slides, Inova Diagnostics, USA. Order No: 508100.20) according to the respective manufacturer's protocols and as recommended by current guidelines.[4] The initial dilution was 1:20 and samples were further diluted up to 1:320 on rodent substrates and 1:640 on HEp-2 cells. SMA staining patterns were assessed using a fluorescence microscope (Olympus BX60 microscope, Evident Europe GmbH, Germany) as published.[17]

Assessment of autoantibodies via ELISA

All ELISA testing was performed centrally at Hannover Medical School, Germany, and the observers were blinded to diagnosis or any clinical data. The presence of F-actin antibodies was assessed using a commercial ELISA (Quanta Lite Actin IgG, Inova Diagnostics, USA, order number 708785) according to the manufacturer's instructions. Commercial ELISAs from three different manufacturers were used to detect ANA according to the manufacturer's instructions (Quanta Lite ANA ELISA, Inova Diagnostics Inc., San Diego, CA, USA, order number 708750; ANA Screening Test, Bio-Rad Laboratories, Inc., Hercules, CA, USA, order number 96AN; ANA Screen ELISA, Euroimmun Medizinische Labordiagnostika AG, Lübeck, Germany, order number EA 1590-9601-11 G). The presence of pIgG was tested by a custom ELISA using BSA as the blocking reagent and HIP1R as the autoantigen in a single 1:100 dilution, as previously published.[9]

Statistical analysis

Continuous variables are expressed as median and range and categorical variables as numbers and percentages. The Chi^2 test was used to compare categorical data between two groups and the Mann-Whitney *U* test was used to compare quantitative data between two groups. The Kruskal-Wallis test was used to compare quantitative data between more than

two groups. The diagnostic test accuracy was calculated as (true positive + true negative)/total number. The AUC and Youden's index were used to determine cut-off values. AUCs were compared using DeLong's test with Bonferroni's method to adjust for multiple comparisons.[18,19] Contingency tables were used to compare the results of two tests. Inter-rater reliability between two tests was analysed using Cohen's kappa coefficient (κ) with the Psych package in R statistical software (version 4.1.2, R Core Team).[20] To simplify the interpretation of the κ coefficient, a κ ≤0.60 was interpreted as inadequate or low agreement, a κ between 0.61 and 0.79 as moderate agreement, and a κ ≥0.80 as high agreement.[21] *p* values below 0.05 (two-tailed) were considered significant in all analyses. Statistical analysis was performed using SPSS version 22.0 (SPSS, Chicago, USA), GraphPad Prism (version 6, Boston, USA) and R Statistical Software (version 4.1.2, R Core Team). There were no missing data for the serological tests used and no equivocal results.

Results

Sixty-nine children with untreated AIH, 13 children with AISC and 120 children with non-AIH-LD were included in the current study (Fig. 1). AIH and AISC were merged into one group, because the discrimination of AIH and AISC can be challenging and the diagnosis of AISC is sometimes made during follow-up as the disease progresses. The blood samples of all groups were taken at the timepoint of first diagnosis, exclusively before initiation of immunosuppression. The median age was similar between all groups (12 [2-17] *vs.* 12 [0-17]; *p* = 0.967). Children with AIH/AISC were more likely to be female than non-AIH-LD controls (58% *vs.* 41%; *p* = 0.013). Median transaminase and IgG levels were higher in children with AIH/AISC (*p* <0.001 for all variables). Children with AIH/AISC more often had concomitant autoimmune diseases and were positive for autoantibodies during their original testing at diagnosis (Table 1 and Table S1).

IFT-based autoantibody testing: ANA

We performed IFT for the detection of ANA on rodent tissue sections and HEp-2 cells. Sensitivity, specificity and accuracy for the prediction of AIH/AISC were dependent on titre on both rodent tissue and HEp-2 cells, with decreasing sensitivity and increasing specificity at higher dilutions (Table 2). At the lowest dilution (1:20), sensitivity was 86.6% for ANA on rodent tissue sections and 92.7% for ANA on HEp-2 cells, whereas specificity was low at 26.7% on rodent tissue sections and 18.3% on HEp-2 cells. Accuracy for AIH/AISC was comparable for the detection of ANA on rodent tissue sections and HEp-2 cells (51.0% *vs.* 48.5% at 1:20). Accuracy was highest at a titre of

Table 1. Demographics of the cohort.

	AILD (n = 82)	Non-AIH-LD (n = 120)	
	Median (min; max)/n (%)	Median (min; max)/n (%)	*p* values
Age [years]	12 (2; 17)	12 (0;17) (n = 118/120)	0.967
Sex, male	34 (42)	71 (59)	0.013
ALT [xULN]	8.7 (0.4; 84.9) (n = 69/82)	1.8 (0.2; 216.6) (n = 109/120)	0.000
AST [xULN]	10.0 (0.5; 114.1)	1.5 (0.3; 152.4) (n = 110/120)	0.000
ALP [xULN]	1 (0.2; 6.6) (n = 78/82)	1 (0.3; 3.1) (n = 77/120)	0.020
GGT [xULN]	2.1 (0.2; 15.5) (n = 81/82)	1 (0.1; 22.9) (n = 112/120)	0.000
Bilirubin [xULN]	1.6 (0.2; 23.2) (n = 80/82)	0.6 (0.1; 60.4) (n = 109/120)	0.000
IgG [xULN]	1.5 (0.5; 6.1) (n = 80/82)	0.7 (0.3; 10.0) (n = 82/120)	0.000
IgG elevated, yes	65 (81)	12 (15)	0.000
HIP1R [normalized abitrary units]	1.73 (0.33; 3.76)	0.96 (0.1; 2.8)	0.000
Center			
Hanover	48 (59)	44 (37)	0.000
London	21 (26)	21 (18)	
Istanbul	6 (7)	20 (17)	
Other	7 (9)	35 (29)	
Coexisting autoimmune diseases			
Overall	11 (13)	1 (0)	0.012
None	69 (86)	119 (99)	
Hennoch Schonlein purpura	1 (1)	0 (0)	
Grave's disease	1 (1)	0 (0)	
Type 1 diabetes	3 (4)	0 (0)	
Inflammatory bowel disease	4 (5)	0 (0)	
Hashimoto's disease	0 (0)	1 (1)	
Celiac disease	1 (1)	0 (0)	
Antibody positivity per local testing			
Any	74 (90)	69 (68)	0.000
ANA	54 (66)	39 (39)	0.000
SMA	57 (70)	38 (38)	0.000
LKM	5 (8)	0 (0)	0.010
LC1	1 (10)	0 (0)	0.052
SLA	2 (4)	0 (0)	0.090

AIH, autoimmune hepatitis; AILD, autoimmune liver disease; AISC, autoimmune sclerosing cholangitis; ALP, alkaline phosphatase; ALT, alanine aminotransferase; ANA, antinuclear antibodies; AST, aspartate aminotransferase; GGT, gamma-glutamyltransferase; IgG, immunoglobulin G; LC1, anti-liver cytosol type 1 antibodies; LKM1, anti-liver kidney microsomal type 1 antibodies; non-AIH-LD, non-AIH, non-AISC liver disease; SLA, soluble liver antigen; SMA, anti-smooth muscle antibodies; xULN, times the upper limit of normal.
Mann-Whitney *U* test.

Table 2. Test performance of IFT-based autoantibody detection in treatment-naïve juvenile AIH/AISC *vs.* non-AIH-LD.

	Titer	Sensitivity	Specificity	PPV	NPV	Accuracy
**ANA IFT**						
Any tissue positivity	1:20	86.6%	26.7%	44.7%	74.4%	51.0%
	1:40	86.6%	27.5%	44.9%	75.0%	51.5%
	1:80	84.2%	38.3%	40.6%	78.0%	56.9%
	1:160	72.0%	65.8%	59.0%	77.5%	68.3%
	**1:320**	**57.3%**	**85.0%**	**72.3%**	**74.5%**	**73.8%**
	1:640	32.9%	94.2%	79.4%	67.3%	69.3%
HEp-2 cell positivity	1:20	92.7%	18.3%	43.7%	78.6%	48.5%
	1:40	92.7%	20.0%	44.2%	80.0%	49.5%
	1:80	90.2%	30.0%	46.8%	81.8%	84.5%
	1:160	75.6%	60.8%	56.9%	78.5%	66.8%
	**1:320**	**56.1%**	**85.0%**	**71.9%**	**73.9%**	**73.3%**
	1:640	31.7%	93.3%	76.5%	66.7%	68.3%
**SMA IFT**						
Any SMA	1:20	90.2%	15.8%	40.6%	42.3%	46.0%
	1:40	89.2%	17.5%	42.4%	70.0%	46.5%
	1:80	76.8%	29.2%	42.6%	64.8%	48.5%
	1:160	61.0%	74.2%	61.7%	73.6%	68.8%
	**1:320**	**45.1%**	**95.0%**	**86.1%**	**71.7%**	**74.8%**
	1:640	30.5%	99.2%	96.2%	67.6%	71.3%
V	1:20	13.4%	75.0%	26.8%	55.9%	50.0%
	1:40	13.4%	75.0%	26.8%	55.9%	50.0%
	1:80	11.0%	79.2%	26.5%	56.5%	51.5%
	1:160	3.7%	92.5%	25.0%	58.4%	56.4%
	1:320	1.2%	98.3%	33.3%	59.3%	58.9%
	**1:640**	**0.0%**	**100.0%**		**59.4%**	**59.4%**
VG	1:20	45.1%	70.0%	50.7%	65.1%	59.9%
	1:40	43.9%	71.7%	51.4%	65.2%	60.4%
	1:80	40.2%	76.7%	54.1%	65.3%	61.9%
	1:160	39.0%	93.3%	80.0%	69.1%	71.3%
	**1:320**	**32.9%**	**99.2%**	**96.4%**	**68.4%**	**72.3%**
	1:640	24.4%	100.0%	100.0%	65.9%	69.3%
VGT	1:20	11.0%	100.0%	100.0%	62.2%	63.9%
	1:40	11.0%	100.0%	100.0%	62.2%	63.9%
	1:80	11.0%	100.0%	100.0%	62.2%	63.9%
	**1:160**	**11.0%**	**100.0%**	**100.0%**	**62.2%**	**63.9%**
	1:320	9.8%	100.0%	100.0%	61.9%	63.4%
	1:640	9.8%	100.0%	100.0%	61.9%	63.4%

AIH, autoimmune hepatitis; AISC, autoimmune sclerosing cholangitis; ANA, antinuclear antibodies; IFT, immunofluorescence testing; non-AIH-LD, non-AIH, non-AISC liver disease; NPV, negative predictive value; PPV, positive predictive value; SMA, anti-smooth muscle antibodies; V, staining of vessels; VG, staining of vessels and glomeruli; VGT, staining of vessels, glomeruli and tubuli.
The titer with the highest diagnostic accuracy is marked in bold.

1:320 on both rodent tissue sections and HEp-2 cells (73.8% and 73.3%, respectively) (Table 2).

The two most common staining patterns of nuclei on HEp-2 cells were fine speckled (AC-4 [39%] according to the international consensus on ANA patterns[22,23]) and homogeneous (AC-1 [51%]) in children with AIH/AISC with a homogeneous staining pattern more common in AIH/AISC and a fine speckled pattern more common in non-AIH-LD (Table S2).

The diagnostic accuracies of ANA by IFT for predicting AIH (excluding AISC cases) *vs*. non-AIH-LD are outlined in Table S3 and were comparable to those observed in the overall cohort (75.7% for ANA on rodent tissue sections and 75.1% for ANA on HEp-2 cells both at 1:320).

IFT-based autoantibody testing: SMA

As with ANA, sensitivity for the prediction of AIH/AISC decreased and specificity increased at higher SMA titres (Table 2). At the lowest dilution (1:20), sensitivity was 90.2%, specificity was 15.8% and accuracy was 46.0% for any SMA positivity. Accuracy was highest (74.8%) at a titre of 1:320 with a sensitivity of 45.1% and a specificity of 95.0% for any SMA positivity. SMA can stain smooth muscle in different compartments on rodent tissue sections, *e.g.* vessels (v), glomeruli (g) and tubules (t). SMA staining patterns on rodent tissue sections helped to increase specificity and accuracy. While the V pattern (staining vessels only) was associated with the lowest accuracy, VG (vessels and glomeruli) and especially VGT (vessels, glomeruli, tubules) showed higher accuracies (Table 2), similar to adults,[5] with the VGT pattern being the most specific.

The diagnostic accuracies of SMA staining on rodent tissue for predicting AIH (excluding AISC cases) *vs.* non-AIH-LD are outlined in Table S3 and were comparable to those in the overall cohort.

ELISA-based detection of ANA, F-actin and pIgG

We evaluated the diagnostic value of commercial ELISA-based autoantibody tests to discriminate between AIH/AISC and non-AIH-LD (Table 3). For ANA detection, sensitivities ranged from 23.2% (Euroimmun) to 62.2% (Inova) using the cut-offs

Table 3. Test performance of ELISA-based autoantibody detection in treatment-naïve juvenile AIH/AISC *vs.* non-AIH-LD.

	ANA						
**Recommended cut offs**	**Euroimmun**	**Bio-Rad**	**Inova**		**F-actin**		**HIP1R/BSA pIgG**
Cut-off	≥1.0	≥1.0	>20(-60)	>60	>20 (-30)	>30	1.27 nAU
Sensitivity	23.2%	61.0%	62.2%	20.7%	74.4%	58.5%	73.2%
Specificity	90.8%	80.0%	69.2%	94.2%	80.0%	94.2%	86.7%
PPV	63.3%	67.6%	58.0%	70.8%	71.8%	87.3%	79.0%
NPV	63.4%	75.0%	72.8%	63.5%	82.1%	76.9%	82.5%
Accuracy	63.4%	72.3%	66.3%	64.4%	77.7%	79.7%	81.2%

AIH, autoimmune hepatitis; AISC, autoimmune sclerosing cholangitis; ANA, antinuclear antibodies; pIgG, polyreactive immunoglobulin G; non-AIH-LD, non-AIH, non-AISC liver disease; NPV, negative predictive value; PPV, positive predictive value.

provided by the respective manufacturers. Specificity was lowest for Inova (69.2%) at their lower proposed cut-off and highest for Inova at their second, higher proposed cut-off (94.2%) while Euroimmun provided the highest specificity at a single cut-off (90.8%). The overall accuracy ranged from 63.4% (Euroimmun) to 72.3% (Bio-Rad). ELISA kits containing HEp-2 extracts (Bio-Rad, Inova) had higher sensitivities, whereas specificity was highest for Euroimmun at a single cut-off and Inova with a cut-off aiming to maximize specificity.

The commercial ELISA for F-actin had sensitivities of 74.4% and 58.5%, and specificities of 80.0% and 94.2% at the two cut-offs (20 and 30 relative units) provided by the manufacturer, while the accuracy was 77.7% and 79.7% (Table 3). Finally, the custom-made pIgG ELISA had a sensitivity of 73.2%, a specificity of 86.7% and an accuracy of 81.2% when using the originally identified cut-off[9] (Table 3).

Local cut-offs were generated for all ELISAs as recommended by the manufacturers. However, the accuracies for the diagnosis of AIH/AISC in this cohort of children with liver

A

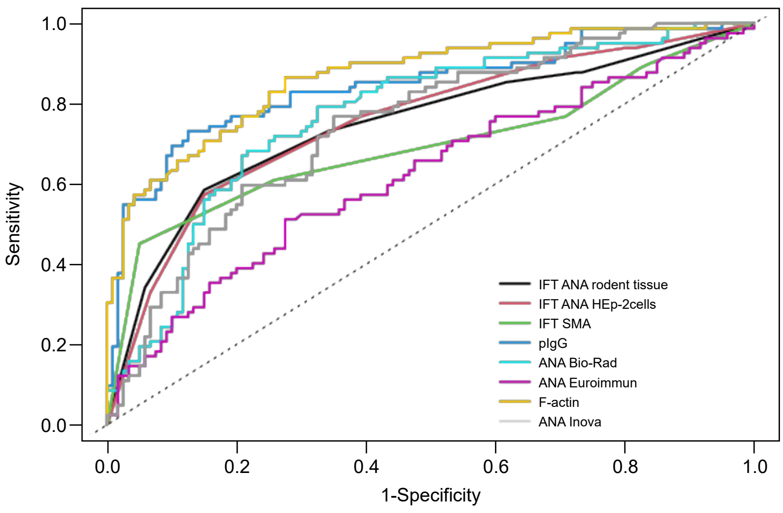

B

		AUC	95% CI Lower Limit	95% CI Upper Limit
IFT	IFT ANA rodent tissue	0.748	0.677	0.819
	IFT SMA	0.694	0.615	0.773
	IFT ANA Hep-2	0.756	0.688	0.824
ELISA	F-actin ELISA	0.868	0.818	0.919
	PIgG	0.844	0.786	0.902
	ANA Bio-Rad	0.772	0.706	0.838
	ANA Inova	0.745	0.677	0.813
	ANA Euroimmun	0.622	0.542	0.702

C

		Adjusted *p*
IFT ANA rodent tissue	IFT SMA	1.000
	IFT ANA HEp-2 cells	
	pIgG	0.192
	F-actin	1.000
	ANA Inova	0.576
	ANA Euroimmun	
	ANA Bio-Rad	1.000
IFT SMA	IFT ANA HEp-2 cells	1.000
	pIgG	0.074
	F-actin	0.007
	ANA Inova	1.000
	ANA Euroimmun	
	ANA Bio-Rad	
IFT ANA HEp-2 cells	pIgG	1.000
	F-actin	0.265
	ANA Inova	1.000
	ANA Euroimmun	0.339
	ANA Bio-Rad	1.000
pIgG	F-actin	1.000
	ANA Inova	0.833
	ANA Euroimmun	<0.001
	ANA Bio-Rad	1.000
F-actin	ANA Inova	0.121
	ANA Euroimmun	<0.001
	ANA Bio-Rad	0.649
ANA Inova	ANA Euroimmun	0.598
	ANA Bio-Rad	1.000
ANA Euroimmun	ANA Bio-Rad	0.130

Fig. 2. Test performance of different assays for the diagnosis of treatment-naive juvenile AIH/AISC. (A,B) ROC curves including the corresponding AUCs and 95% CIs for the diagnosis of AIH/AISC by different assays. (C) DeLong test with adjustment for multiple comparisons with the Bonferroni method indicating the *p* value for the respective comparisons of AUCs between assays. AIH, autoimmune hepatitis; AISC, autoimmune sclerosing cholangitis; ANA, antinuclear antibodies; HEp-2, human epithelioma-2; IFT, immunofluorescence testing; pIgG, polyreactive immunoglobulin G; SMA, anti-smooth muscle antibodies.

disease could not be significantly improved by adjusted ELISA cut-offs (accuracies: ANA ELISA: 63.9%-73.8%; F-actin 80.2%; pIgG: 81.2%).

The diagnostic accuracies of the autoantibody ELISAs for predicting AIH (excluding AISC cases) *vs*. non-AIH-LD are outlined in Table S4 and were comparable to those in the overall cohort.

Comparison of ELISA- and IFT-based detection of autoantibodies

The diagnostic accuracy for identifying juvenile AIH/AISC was comparable between the different tests (Fig. 2). The AUCs of IFT were 0.748 (ANA detection on rodent tissue sections), 0.756 (ANA detection on HEp-2 cells), and 0.694 (any SMA on rodent tissue sections). The AUCs of the ANA ELISAs were 0.622 (Euroimmun), 0.745 (Inova) and 0.772 (Bio-Rad) with no difference between the three (Fig. 2A-C). The AUCs for IFT-based detection of ANA were not different from the AUCs for ELISA-based detection (Fig. 2C).

The highest AUCs were achieved by F-actin ELISA (0.868) and pIgG ELISA (0.844; Fig. 2A,B), with no difference between the two (Fig. 2C). The F-actin and pIgG ELISAs were the only tests with a better AUC than ANA detection by the Euroimmun ELISA (Fig. 2C).

The findings were comparable if only AIH cases were compared to non-AIH-LD controls (Fig. S2).

Age-related autoantibody concentrations and titres

Current guidelines recommend lower cut-off values for IFT in children compared to adults (1:20 *vs*. 1:40).[1] To assess age-dependent autoantibody titres or concentrations, the cohort was split at the median age (12 years) to investigate the effect of age on the distribution of autoantibodies in juvenile AIH/AISC. The distribution of autoantibodies, as well as their titres or concentrations, did not differ between children in either group (Fig. 3). There were no significant differences between the two age groups in any test when autoantibody frequencies (positive/negative or titres) were analysed (Fig. 3A). Similarly,

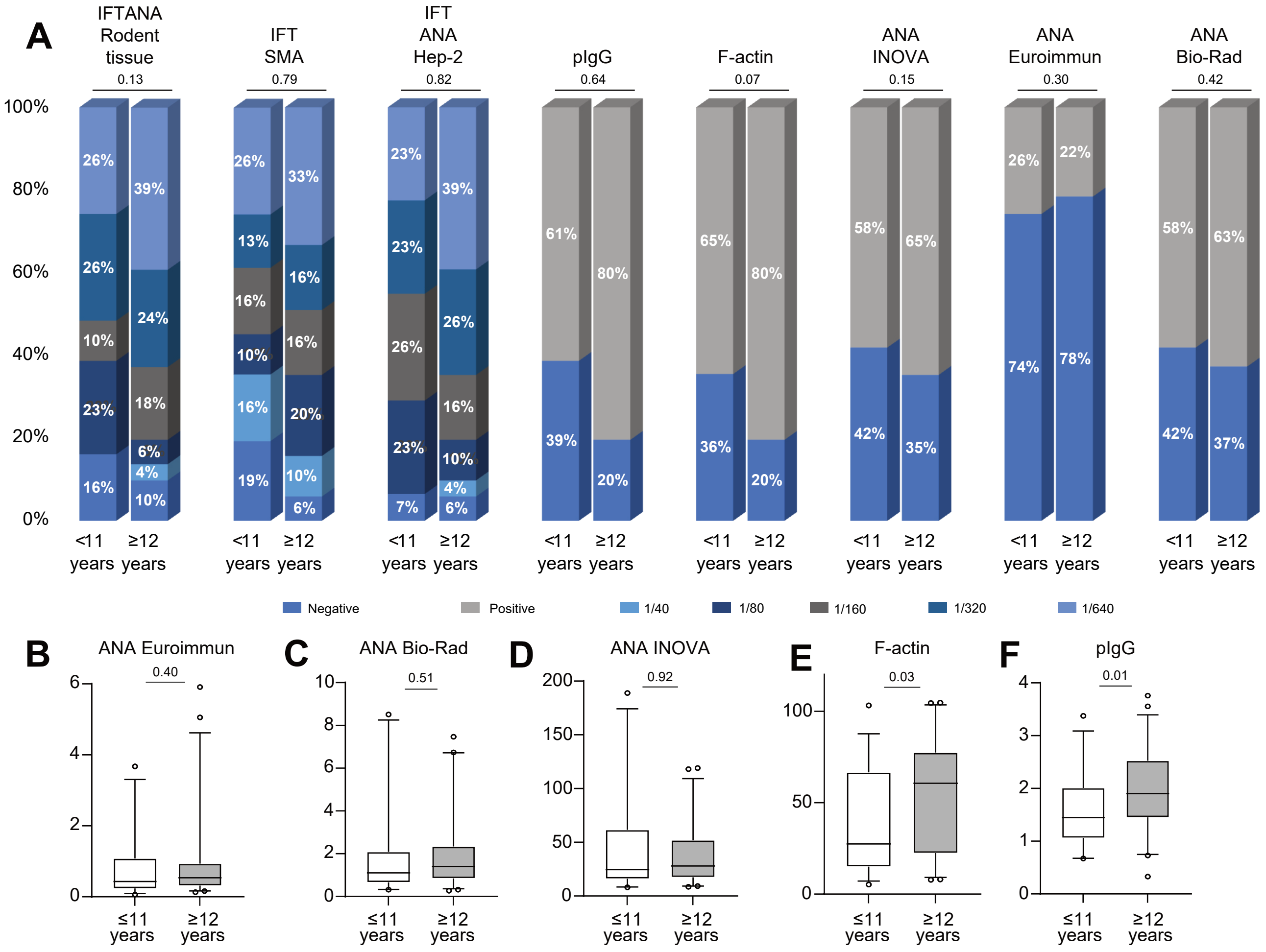

Fig. 3. Distribution of antibodies in children with treatment-naive AIH/AISC according to age. Frequencies of positive or negative test results (ELISA-based assessment) or respective titres (IFT-based assessment) shown as stacked bars in children with AIH younger (≤11) or older (≥12) than the median age of the cohort. Chi^2 test (A). Levels of antibodies shown as box plots with 95% CIs as assessed by ELISA dependent on the age of the patients (B: ANA ELISA Euroimmun; C: ANA ELISA Bio-Rad; D: ANA ELISA Inova; E: F-actin ELISA; F: pIgG ELISA); Mann-Whitney *U* test. AIH, autoimmune hepatitis; AISC, autoimmune sclerosing cholangitis; ANA, antinuclear antibodies; HEp-2, human epithelioma-2; IFT, immunofluorescence testing; nAU, normalized arbitrary units; pIgG, polyreactive immunoglobulin G; RU, relative units; SMA, anti-smooth muscle antibodies.

autoantibody concentrations were not age dependent for all ANA ELISAs (Fig. 3B-D). However, concentrations of anti-F-actin and pIgG were higher in older children (Fig. 3E,F), without affecting the frequencies of positive and negative findings (Fig. 3A).

Agreement between different test systems

For the whole cohort, the agreement between ELISA-based ANA detection and IFT-based ANA detection was low, whereas the agreement between IFT substrates was moderate to strong (κ 0.71 at 1:20 and κ 0.81 at 1:320) (Table S3): Of 71 children with AIH/AISC who tested positive for ANA by IFT on rodent tissue sections, Euroimmun, Inova and Bio-Rad ELISAs detected ANA in 18/71 (25.4%), 45/71 (63.4%) and 47/71 (66.2%), respectively. Eleven children with AIH/AISC tested negative by IFT on rodent tissue sections. One of these cases was positive by Euroimmun ELISA (9.1%), 6/11 (54.6%) by Inova ELISA and 3/11 (27.3%) by Bio-Rad ELISA. The agreement between ANA ELISAs and IFT on HEp-2 cells was comparable to IFT on rodent tissue sections.

The agreement between the various autoantibody test methodologies regarding dichotomous results (positive or negative) are summarized in Table S5. The agreement between ANA determined via IFT on rodent tissue sections as gold standard and HEp-2 cells was moderate to strong (κ 0.71-0.81). The agreement between the ANA ELISAs and ANA on IFT rodent tissue sections was low for high titres (≥1:320: κ 0.19-0.41) and non-existent for low titres (≥1:20: κ <0.10). Similar results were found for the agreement of F-actin and pIgG ELISAs with the gold standard of ANA IFT on rodent tissue sections and F-actin with SMA on rodent tissue sections (Table S5).

The presence of pIgG and F-actin antibodies was independent in juvenile AIH/AISC (p = 0.013; Fig. S1: κ 0.45 in Table S3). PIgG positivity was 52% in F-actin-negative patients. The sensitivity of IFT was unaffected by sample storage duration, whereas specificity was lower in samples with shorter storage. In contrast, ELISA sensitivity was higher in older samples, while specificity remained unchanged (Fig. S3; Table S6). Correlation with total IgG levels was only weak to moderate for all tests (Table S7).

Discussion

Our multicentre study is the first head-to-head comparative evaluation of IFT- and ELISA-based autoantibody detection for the diagnosis of AIH and AISC in a large, unique, multinational juvenile liver disease cohort.

Adding to a single available study in adults,[5] we demonstrate that in juvenile AIH and AISC, autoantibodies – namely ANA and SMA – can be detected not only by IFT on rodent tissue sections, but also using HEp-2 cells for ANA detection or ANA ELISA with comparable test performance, depending on the specific assay used for ANA ELISA. Further validation is needed to determine the value of ANA staining patterns for the diagnosis of AIH/AISC, as homogeneous staining of nuclei is also detected at a relevant frequency in children with liver diseases other than AIH/AISC in our study. We also found a higher sensitivity (92.7%) at lower titres on HEp-2 cells, which favours its use as a screening test.[5] As demonstrated in paediatric rheumatic disease and supporting our findings, specificity is low when used as a screening test, especially in a population with a low pre-test probability and likelihood of overt (rheumatic) autoimmune disease.[24–26] A positive ANA with a titre of at least 1:80 is also observed in 43% of children with metabolic liver disease.[27] Our results, in conjunction with the literature, advocate caution in the interpretation of ANA findings on IFT, especially at very low titres (1:20), which are currently considered positive within the scoring system,[1] and highlight the need to place these findings in a precise clinical context to achieve a diagnosis of AIH/AISC in children when investigating an unknown liver disease.

We observe similar results for the detection of SMA by IFT. While it has a high sensitivity at a low titre of 1:20, the specificity is low for SMA when the VGT staining pattern is not incorporated. In agreement with the literature,[5,28,29] positivity for SMA without specific attention to the VGT staining pattern must be interpreted with caution in the work-up of suspected juvenile AIH and AISC, and specific SMA staining patterns should be taken into account.

In the work-up of rheumatic diseases, screening for ANA by IFT on HEp-2 cells is considered the gold standard, while the widespread use of automated, antigen-specific methods, *e.g.* ELISA, is recognised and their performance was evaluated more than 20 years ago in comparison with IFT in patients with rheumatic diseases.[30–32] These studies, as well as ours, highlighted the heterogeneity of test performance depending on the nuclear antigens used by the respective manufacturers.[5,31,32] Our data in children with liver disease support previous findings in patients with rheumatic disease[31,32] and adult patients with liver disease[5] of heterogeneous test performance between different manufacturers of ANA ELISAs, as well as robust test performance for screening purposes that was not inferior to IFT-based detection on rodent tissue sections or HEp-2 cells in our cohort. Our data suggest that caution should be exercised in interpreting the results of ANA detection by ELISA, depending on whether the test is designed to be specific (*i.e.* Euroimmun using a defined set of nuclear antigens) or more balanced (Bio-Rad and Inova assays using nuclear extracts). Nevertheless, our data suggest that ELISAs with locally validated cut-offs have good accuracy in detecting ANA in the work-up of liver disease in children. Although the accuracies of ANA IFT and ANA ELISA, or SMA IFT and F-actin ELISA, were similar, the actual level of agreement between them was relatively low. This is because AIH/AISC cases may be detected by one assay or the other without both necessarily being positive, as exemplified in Fig. S1. The discrepancy was more pronounced for low autoantibody titres. This is consistent with recent findings in adults with autoimmune and non-autoimmune liver diseases.[5] Although the various assays contain similar antigens or antigen mixtures, the assay methodology (IFT or ELISA) seems to be relevant. Therefore, different methodologies complement rather than substitute each other. The best AUCs in our study for predicting or excluding juvenile AIH or AISC were achieved by the detection of F-actin and pIgG by ELISA, demonstrating at least non-inferiority to all other tests. The high performance of both tests in predicting juvenile AIH/AISC is in agreement with previous studies for which we provide validation with our study.[5,9,10,33–35]

We provide evidence that the serological likelihood of AIH and AISC should not only be determined by the assessment of

ANA and SMA by IFT on rodent tissue sections, as previously advocated, but also by IFT on HEp-2 cells and ELISA-based systems, including F-actin and pIgG. While the use of varying assay systems is common in real-world clinical practice, we provide the first evidence for this practice in children. Due to the retrospective nature of this study, the histological criteria for the presence of AIH were not evaluated on the basis of the most recent recommendations.[36] An updated juvenile scoring system, integrating the latest advances in autoantibody and histopathological evaluation, promises to improve the diagnostic certainty of AIH and AISC in children.

Our study has several strengths. Sera from children with AIH, AISC and a heterogeneous variety of other liver diseases were tested centrally using the same methods by technicians blinded to any further information, in contrast to a single available comparative study in adults using locally derived IFT results, as well as a more homogenous comparator cohort.[5] Our study includes samples from expert centres in eight European countries, ensuring representativeness of the paediatric population in Europe. Although juvenile AIH/AISC is rare and few centres consistently archive biomaterial from such patients for studies such as ours, our cohort provides a good sample size to address the issue of serological testing in this population. We also show that autoantibody frequencies and median levels mostly did not differ by age in a well-characterised cohort of children with AIH, albeit with a small number of very young children which precluded a more detailed analysis (*e.g.* by dividing the cohort into quartiles). Pending prospective validation, this challenges the current notion that an ANA and SMA detected at a low titre is necessarily indicative of autoimmune liver disease, as previously shown in children with metabolic dysfunction-associated steatotic liver disease.[27] Therefore, clinicians should be aware that the high sensitivity at low autoantibody titres (1:20–1:40) is accompanied by low specificity, and that the optimal accuracies for ANA and SMA (including V and VG staining patterns) in IFT (on rodent tissue and HEp-2 cells) were observed at a titre of 1:320.

Our study also has limitations. The retrospective design meant that samples were stored until use in this study, which is subject to all the limitations inherent to a retrospective study design. The representativeness of our study is lower for AISC due to the smaller number of available sera. So, the AIH and AISC samples were merged because it can be difficult to distinguish between the two, and this is sometimes only possible at a later stage of the disease. In addition, AISC may develop from AIH at later disease stages, thereby limiting diagnostic certainty at any time during a patients disease course. However, we also outlined the test results for AIH without AISC to present the data as comprehensively as possible. It has been shown that frozen storage of serum samples does not affect the levels of thyroid-related autoantibodies.[37] Although this was not completely transferable to our study, all samples were tested at the same time in Hannover. Therefore, the comparison of the tests with each other should remain valid. However, long storage durations might lead to an overestimation of the assay sensitivity in ELISAs. For pIgG, we have shown increasing levels over time, which was accounted for in the original publications by normalisation to similarly stored control samples.[9,10] The quantification of IgG autoantibodies is biased by the overall level of IgG although there is only weak to moderate correlation between IgG levels and antibody titres or levels in our study. We have previously demonstrated preserved reactivity for pIgG even when samples were further diluted, which would reflect a normalisation of IgG concentrations for most cases analysed.[9] We ultimately did not normalise each sample for IgG concentrations because this is not part of routine clinical practice, and the study should reflect real-world practice. Generalisability is further limited by the lack of patient samples from outside Europe. Therefore, future validation studies should include centres from other continents, including children from different ethnic backgrounds, to determine the influence of geographical and ethnic variation on the assays detecting autoantibodies. Similarly, sex differences that may affect autoantibody frequencies, *i.e.* a female predominance inherent to AIH, were not considered in the analysis, although a higher frequency of autoantibodies in females will certainly not affect the comparative analysis between assays.

In conclusion, our study in a representative European cohort of children with liver disease provides evidence for the use of not only rodent tissue sections but also HEp-2 cells for the detection of ANA in the work-up of suspected juvenile AIH and AISC. Although juvenile AIH itself is rare, our findings apply to the broader clinical scenario of the work-up of any non-viral liver disease. Our data suggest that ELISA may complement but not fully substitute IFT. In addition, F-actin ELISA shows at least non-inferior performance to any SMA positivity by IFT on rodent tissue sections, supporting its implementation in the scoring system to determine the likelihood of juvenile AIH. In addition, pIgG is complementary to the other autoantibodies and might be implemented in the work-up of suspected AIH and AISC in the future as suggested by the most recent European AIH guideline.[3] While assays for the detection of autoantibodies need to be locally validated and specifically selected for their respective use, our data support the complementary use of detection systems in addition to rodent tissue sections as current gold standard. Finally, the good diagnostic performance of all autoantibody methodologies suggests that, after further validation, the current scoring algorithm for juvenile AIH and AISC could accommodate a broader range of assays to better account for the serological heterogeneity of these conditions. A prospective validation of the different autoantibody methods and the revised scoring system for AIH and AISC will be performed in a prospective multicentre study that we have recently initiated (NCT05810480).

Affiliations

[1]*Department of Gastroenterology, Hepatology, Infectious Diseases and Endocrinology, Hannover Medical School, Hannover, Germany;* [2]*Division of Pediatric Gastroenterology and Hepatology, Department of Pediatric Nephrology, Hepatology and Metabolic Disorders, Hannover Medical School, Hannover, Germany;* [3]*Institute of Liver Studies, King's College Hospital; Department of Inflammation Biology, School of Immunology and Microbial Sciences, King's College London, London, United Kingdom;* [4]*Koç University Research Centre for Translational Medicine (KUTTAM) - Liver Immunology Lab, Istanbul, Turkey;* [5]*Department of Gastroenterology, Hepatology, Nutritional Disorders and Pediatrics, The Children's Memorial Health Institute, Warsaw, Poland;* [6]*Department of Medical and Surgical Sciences, University of Bologna, Bologna, Italy;* [7]*Institute of Internal Medicine and Hepatology, Larissa, Greece;* [8]*Department of Medicine and Research Laboratory of Internal Medicine, National Expertise Center of Greece in Autoimmune Liver Diseases, General University Hospital of Larissa, Larissa, Greece;*

[9]Centre for Liver and Gastro Research, National Institute of Health Research Birmingham Biomedical Research Centre, Institute of Immunology and Immunotherapy, The Medical School, Birmingham, United Kingdom & Liver Transplant and Hepatobiliary Unit, University Hospital Birmingham NHS Foundation Trust, Edgbaston, Birmingham, UK; [10]Nantes Université, CHU Nantes, Institut des Maladies de l'Appareil Digestif (IMAD), Hépato-Gastro-Entérologie, Inserm CIC 1413, 44000 Nantes, France; [11]Department of Gastroenterology and Hepatology, Radboud University Medical Center, Nijmegen, The Netherlands; [12]Department of Gastroenterology and Hepatology, Amsterdam University Medical Center, Amsterdam, The Netherlands; [13]Nantes Université, Inserm, Center for Research in Transplantation and Translational Immunology, UMR 1064, F-44000 Nantes, France; [14]European Reference Network on Hepatological Diseases (ERN RARE-LIVER), Hamburg, Germany; [15]PRACTIS & CORE100Pilot Clinician Scientist Programs, Dean's Office for Academic Career Development, Hannover Medical School, Germany

Abbreviations

AIH, autoimmune hepatitis; AISC, autoimmune sclerosing cholangitis; ANA, antinuclear antibodies; HEp-2, human epithelioma-2; IAIHG, International Autoimmune Hepatitis Group; IFT, immunofluorescence testing; IgG, immunoglobulin G; non-AIH-LD, non-autoimmune hepatitis, non-autoimmune sclerosing cholangitis liver disease; pIgG, polyreactive immunoglobulin G; SMA, anti-smooth muscle antibodies.

Financial support

BE was supported by the PRACTIS – Clinician Scientist Program of Hannover Medical School, funded by the German Research Foundation (DFG, ME 3696/3) and by a bridging program as part of the CORE100Pilot for clinician scientists in transplantation medicine, funded by Else Kröner Fresenius Stiftung (2020_EKSP.78) and the Ministry for Science and Culture of Lower Saxony (ZN3720). Inova Diagnostics Inc. and Euroimmun Medizinische Labordiagnostika AG provided ELISAs free of charge for this project. Inova Diagnostics Inc. and Euroimmun Medizinische Labordiagnostika AG provided ELISAs free of charge. YHO received funding from the Sir Jules Thorn Charitable Trust and the Whitney-Wood Scholarship of the Royal College of Physicians.

Conflict of interest

RT is co-inventor of the patent for the use of polyreactive IgG for the diagnosis of AIH (patent number: EP3701264 B1; US 12,044,682 B2). All other authors have nothing to declare regarding this paper.

Please refer to the accompanying ICMJE disclosure forms for further details.

Authors' contributions

Study concept and design: BE, RT. Acquisition of data: TK, NJ, NH, SL, MY, WJ, CL, KZ, YHO, JG, SP, JPHD, AR, GND, LM, PS, CR, YM, HW, UB, BE, RT. Analysis and interpretation of data: TK, BE, RT. Drafting of the manuscript: TK, BE, RT. Critical revision of the manuscript for important intellectual content: NJ, NH, SL, MY, WJ, CL, KZ, YHO, JG, SP, JPHD, AR, GND, LM, PS, CR, YM, HW, UB. Statistical analysis: TK, BE. Obtained funding: HW, BE, RT. Administrative, technical, or material support: NJ, NH, SL, MY, WJ, CL, KZ, YHO, JG, SP, JPHD, AR, GND, LM, PS, CR, YM, HW, UB, BE, RT. Study supervision: BE, RT.

Declaration of generative AI and AI-assisted technologies in the writing process

During the preparation of this work the author(s) used DeepL Write in order to improve use of the English language. After using this tool, the authors reviewed and edited the content as needed and take full responsibility for the content of the publication.

Data availability

The data of this study are available from the corresponding authors upon reasonable request.

Supplementary data

Supplementary data to this article can be found online at https://doi.org/10.1016/j.jhepr.2025.101706.

References

Author names in bold designate shared authorship

[1] Mieli-Vergani G, Vergani D, Baumann U, et al. Diagnosis and management of pediatric autoimmune liver disease: ESPGHAN hepatology committee position statement. J Pediatr Gastroenterol Nutr 2018;66:345–360.

[2] Mack CL, Adams D, Assis DN, et al. Diagnosis and management of autoimmune hepatitis in adults and children: 2019 practice guidance and guidelines from the American Association for the study of liver diseases. Hepatology 2019. https://doi.org/10.1002/hep.31065.

[3] European Association for the Study of the Liver. EASL Clinical Practice Guidelines on the management of autoimmune hepatitis. J Hepatol 2025;83:453–501.

[4] Vergani D, Alvarez F, Bianchi FB, et al. Liver autoimmune serology: a consensus statement from the committee for autoimmune serology of the International Autoimmune Hepatitis Group. J Hepatol 2004;41:677–683.

[5] **Galaski J**, **Weiler-Normann C**, Schakat M, et al. Update of the simplified criteria for autoimmune hepatitis: evaluation of the methodology for immunoserological testing. J Hepatol 2021;74:312–320.

[6] Klotz W, Herold M. How to test antinuclear antibodies to diagnose autoimmune hepatitis. J Hepatol 2023;79:e206–e207.

[7] Hennes EM, Zeniya M, Czaja AJ, et al. Simplified criteria for the diagnosis of autoimmune hepatitis. Hepatology 2008;48:169–176.

[8] Alvarez F, Berg PA, Bianchi FB, et al. International autoimmune hepatitis group report: review of criteria for diagnosis of autoimmune hepatitis. J Hepatol 1999;31:929–938.

[9] **Taubert R**, **Engel B**, Diestelhorst J, et al. Quantification of polyreactive immunoglobulin G facilitates the diagnosis of autoimmune hepatitis. Hepatology 2022;75:13–27.

[10] **Engel B**, **Diestelhorst J**, Hupa-Breier KL, et al. Detection of polyreactive immunoglobulin G facilitates diagnosis in children with autoimmune hepatitis. Hepatol Int 2024;18:1214–1226.

[11] Engel B, Assis DN, Bhat M, et al. Quo vadis autoimmune hepatitis? - summary of the 5th international autoimmune hepatitis group research workshop 2024. JHEP Rep 2025;7:101265.

[12] Campos-Murguia A, Henjes N, Loges S, et al. Evaluation of immunoserological detection of anti-liver kidney microsomal, anti-soluble liver antigen and anti-mitochondrial antibodies. Scientific Rep 2023;13:10038.

[13] **Socha P**, **Janczyk W**, Dhawan A, et al. Wilson's disease in children: a position paper by the hepatology committee of the European Society for paediatric Gastroenterology, Hepatology and Nutrition. J Pediatr Gastroenterol Nutr 2018;66:334–344.

[14] Vos MB, Abrams SH, Barlow SE, et al. NASPGHAN clinical practice guideline for the diagnosis and treatment of nonalcoholic fatty liver disease in children: recommendations from the expert committee on NAFLD (ECON) and the North American Society of pediatric Gastroenterology, Hepatology and Nu. J Pediatr Gastroenterol Nutr 2017;64:319–334.

[15] European Association for the Study of the Liver. EASL clinical practice guidelines: drug-induced liver injury. J Hepatol 2019;70:1222–1261.

[16] Chapman R, Fevery J, Kalloo A, et al. Diagnosis and management of primary sclerosing cholangitis. Hepatology 2010;51:660–678.

[17] Bottazzo GF, Florin-Christensen A, Fairfax A, et al. Classification of smooth muscle autoantibodies detected by immunofluorescence. J Clin Pathol 1976;29:403–410.

[18] DeLong ER, DeLong DM, Clarke-Pearson DL. Comparing the areas under two or more correlated receiver operating characteristic curves: a nonparametric approach. Biometrics 1988;44:837–845.

[19] Bland JM, Altman DG. Statistics notes: multiple significance tests: the Bonferroni method. BMJ 1995;310:170.

[20] Revelle W. Procedures psychological, psychometric, personality research. 2022.

[21] McHugh ML. Interrater reliability: the kappa statistic. Biochem Med (Zagreb) 2012;22:276–282.

[22] Damoiseaux J, Andrade LEC, Carballo OG, et al. Clinical relevance of HEp-2 indirect immunofluorescent patterns: the International Consensus on ANA patterns (ICAP) perspective. Ann Rheum Dis 2019;78:879–889.

[23] Chan EKL, Damoiseaux J, Carballo OG, et al. Report of the first international consensus on standardized nomenclature of antinuclear antibody HEp-2 cell patterns 2014-2015. Front Immunol 2015;6:412.

[24] Mejdoub S, Hachicha H, Gargouri L, et al. Antinuclear antibodies in children: clinical signification and diagnosis utility. Tunis Med 2021;99:980–984.

[25] McGhee JL, Kickingbird LM, Jarvis JN. Clinical utility of antinuclear antibody tests in children. BMC Pediatr 2004;4:13.
[26] Şahin N, Kısaarslan AP, Çiçek SÖ, et al. Clinical usefulness of anti-nuclear antibody in childhood: real-world experience at a tertiary care center : usefulness of ANA in pediatric autoimmune diseases. Eur J Pediatr 2023;182:3325–3335.
[27] Kohut T, Shah A, Russo P, et al. Autoimmune antibodies in children and adolescents with nonalcoholic fatty liver disease. J Pediatr Gastroenterol Nutr 2022;75:264–268.
[28] Muratori P, Muratori L, Agostinelli D, et al. Smooth muscle antibodies and type 1 autoimmune hepatitis. Autoimmunity 2002;35:497–500.
[29] Granito A, Muratori L, Muratori P, et al. Antibodies to filamentous actin (F-actin) in type 1 autoimmune hepatitis. J Clin Pathol 2006;59:280–284.
[30] **Agmon-Levin N**, **Damoiseaux J**, Kallenberg C, et al. International recommendations for the assessment of autoantibodies to cellular antigens referred to as anti-nuclear antibodies. Ann Rheum Dis 2014;73:17–23.
[31] Gniewek RA, Sandbulte C, Fox PC. Comparison of antinuclear antibody testing methods by ROC analysis with reference to disease diagnosis. Clin Chem 1997;43:1987–1989.
[32] Tonuttia E, Bassetti D, Piazza A, et al. Diagnostic accuracy of ELISA methods as an alternative screening test to indirect immunofluorescence for the detection of antinuclear antibodies. Evaluation of five commercial kits. Autoimmunity 2004;37:171–176.
[33] Frenzel C, Herkel J, Lüth S, et al. Evaluation of F-actin ELISA for the diagnosis of autoimmune hepatitis. Am J Gastroenterol 2006;101: 2731–2736.
[34] Chretien-Leprince P, Ballot E, Andre C, et al. Diagnostic value of anti-F-actin antibodies in a French multicenter study. Ann N Y Acad Sci 2005;1050:266–273.
[35] Maggiore G, Veber F, Bernard O, et al. Autoimmune hepatitis associated with anti-actin antibodies in children and adolescents. J Pediatr Gastroenterol Nutr 1993;17:376–381.
[36] **Lohse AW**, **Sebode M**, Bhathal PS, et al. Consensus recommendations for histological criteria of autoimmune hepatitis from the International AIH Pathology Group. Liver Int 2022;42:1058–1069.
[37] Jensen CZ, Nygaard B, Faber J, et al. Long-term stability of thyroid peroxidase antibody (anti-TPO) in serum in the Danish General Suburban Population Study. Clin Chem Lab Med 2023;61:1590–1596.

Keywords: liver disease; pediatric autoimmune hepatitis; immunofluorescence testing; ELISA; autoantibodies; polyreactive immunoglobulin G.

Received 24 March 2025; received in revised form 24 November 2025; accepted 26 November 2025; Available online 6 December 2025

Journal of Hepatology, Volume *8*

Supplemental information

Complementary use of autoantibody detection methods facilitates diagnosis of juvenile autoimmune hepatitis and autoimmune sclerosing cholangitis

Theresa Kirchner, Norman Junge, Nicole Henjes, Stephanie Loges, Muhammed Yuksel, Wojciech Janczyk, Claudine Lalanne, Kalliopi Zachou, Ye H. Oo, Jérôme Gournay, Simon Pape, Joost PH. Drenth, Amédée Renand, George N. Dalekos, Luigi Muratori, Piotr Socha, Cigdem Arikan, Yun Ma, Heiner Wedemeyer, Ulrich Baumann, Bastian Engel, and Richard Taubert

Complementary use of autoantibody detection methods facilitates diagnosis of juvenile autoimmune hepatitis and autoimmune sclerosing cholangitis

Theresa Kirchner, Norman Junge, Nicole Henjes, Stephanie Loges, Muhammed Yuksel, Wojciech Janczyk, Claudine Lalanne, Kalliopi Zachou, Ye H. Oo, Jérôme Gournay, Simon Pape, Joost P.H. Drenth, Amédée Renand, George N. Dalekos, Luigi Muratori, Piotr Socha, Cigdem Arikan, Yun Ma, Heiner Wedemeyer, Ulrich Baumann, Bastian Engel, Richard Taubert

Table of contents

Fig. S1…………………………………………………………………………….….2

Fig. S2…………………………………………………………………………….….3

Fig. S3……………………………………………………………………………….4

Table S1……………………………………………………………………...….5

Table S2………………………………………………………………….…......5

Table S3………………………………………………………………….…......6

Table S4………………………………………………………………………......7

Table S5………………………………………………………………….…......7

Table S6………………………………………………………………….…......8

Table S7………………………………………………………………….…......9

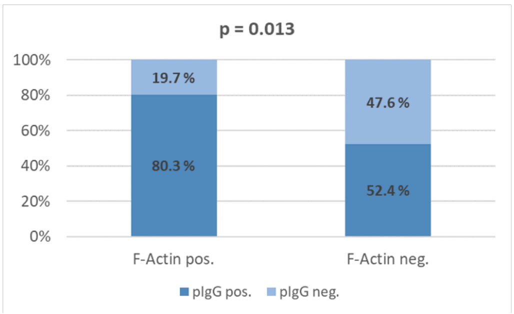

Fig. S1: Frequency of pIgG positive samples dependent on presence of anti-F-actin antibodies

Stacked graphs show frequencies of pIgG positive samples (dark blue) and negative samples (light blue) in children with AIH/AISC with positivity (F-Actin pos.) or negativity (F-Actin neg.) for F-Actin. Chi² test was used for statistical comparison.

A

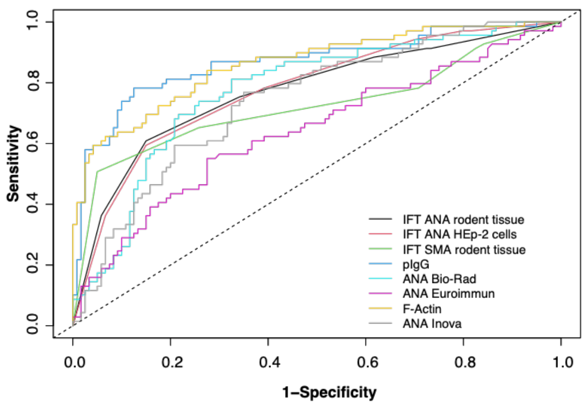

B

		AUC	95% CI	
			LowerLimit	UpperLimit
ELISA	F-Actin ELISA	.861	.804	.917
	PlgG	.866	.807	.925
	ANA Bio-Rad	.774	.704	.843
	ANA Inova	.740	.669	.812
	ANA Euroimmun	.640	.555	.724
IFT	IFT ANA rodent tissue	.771	.699	.843
	IFT SMA	.723	.640	.806
	IFT ANA Hep-2	.777	.709	.806

C

<table>
<tr><th></th><th></th><th>adjusted p</th></tr>
<tr><td rowspan="7">IFT ANA rodent tissue</td><td>IFT SMA</td><td rowspan="5">1.000</td></tr>
<tr><td>IFT ANA HEp-2 cells</td></tr>
<tr><td>plgG</td></tr>
<tr><td>F-Actin</td></tr>
<tr><td>ANA Inova</td></tr>
<tr><td>ANA Euroimmun</td><td>0.576</td></tr>
<tr><td>ANA Bio-Rad</td><td>1.000</td></tr>
<tr><td rowspan="6">IFT SMA</td><td>IFT ANA HEp-2 cells</td><td>1.000</td></tr>
<tr><td>plgG</td><td>0.163</td></tr>
<tr><td>F-Actin</td><td>0.197</td></tr>
<tr><td>ANA Inova</td><td rowspan="3">1.000</td></tr>
<tr><td>ANA Euroimmun</td></tr>
<tr><td>ANA Bio-Rad</td></tr>
<tr><td rowspan="5">IFT ANA HEp-2 cells</td><td>plgG</td><td rowspan="3">1.000</td></tr>
<tr><td>F-Actin</td></tr>
<tr><td>ANA Inova</td></tr>
<tr><td>ANA Euroimmun</td><td>0.376</td></tr>
<tr><td>ANA Bio-Rad</td><td>1.000</td></tr>
<tr><td rowspan="4">plgG</td><td>F-Actin</td><td>1.000</td></tr>
<tr><td>ANA Inova</td><td>0.226</td></tr>
<tr><td>ANA Euroimmun</td><td>< 0.001</td></tr>
<tr><td>ANA Bio-Rad</td><td>1.000</td></tr>
<tr><td rowspan="3">F-Actin</td><td>ANA Inova</td><td>0.275</td></tr>
<tr><td>ANA Euroimmun</td><td>< 0.001</td></tr>
<tr><td>ANA Bio-Rad</td><td>1.000</td></tr>
<tr><td rowspan="2">ANA Inova</td><td>ANA Euroimmun</td><td rowspan="2">1.000</td></tr>
<tr><td>ANA Bio-Rad</td></tr>
<tr><td>ANA Euroimmun</td><td>ANA Bio-Rad</td><td>0.467</td></tr>
</table>

Fig. S2: Test performance of different assays for the diagnosis of treatment-naive juvenile AIH

(A+B) ROC curves including the corresponding AUCs and 95% confidence intervals for the diagnosis of AIH by different assays. (C) DeLong test with adjustment for multiple comparisons with the Bonferroni method indicating the p-value for the respective comparisons of AUCs between assays. IFT: immunofluorescence testing; ANA: antinuclear antibodies; SMA: anti-smooth muscle antibodies; pIgG: polyreactive immunoglobulin G; HEp-2: human epithelioma-2

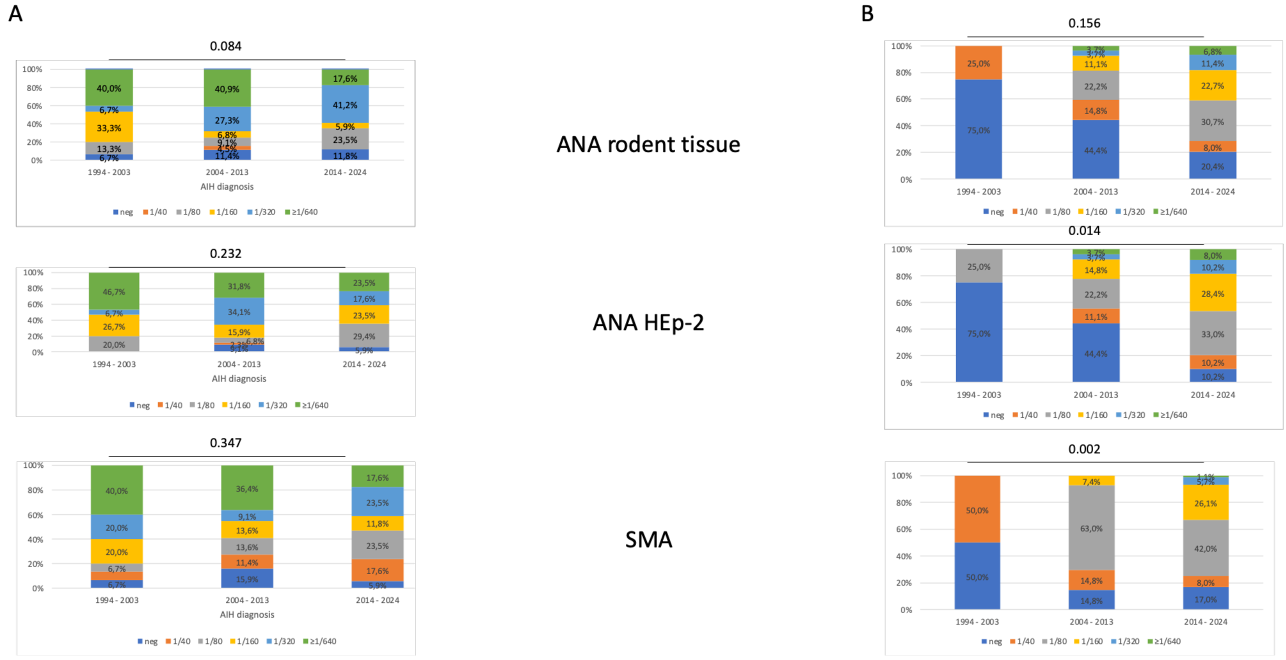

Fig. S3: Frequency of antibody titres in AIH/AISC and controls

Stacked graphs show frequencies of different ANA titres on rodent tissue, HEp-2 cells and SMA titres on rodent tissue in AIH/AISC (A) and non-AIH-LD (B) across three different decades of sample collection. Chi² test was used for statistical comparison.

Table S1: Frequency of non-AIH-non-AISC liver disease controls

n=120	n (%)
MASLD	37 (30.8)
PFIC	8 (6.7)
PSC	13 (10.8)
toxic	4 (3.3)
Wilson's disease	35 (29.22)
alpha 1 antitrypsin deficiency	7 (5.8)
unknown	9 (7.5)
other	7 (5.8)

MASLD: metabolic-dysfunction associated steatotic liver disease; PFIC: progressive familiary intrahepatic cholestasis; PSC: primary sclerosing cholangitis. Other: cytochrome oxidase deficiency, congenital hepatitis with portal hypertension, acute liver failure of unknown aetiology, primary hyperoxaluria, trisomie 21, celiac disease, giant cell hepatitis

Table S2: Staining patterns of ANA on HEp-2 cells

		AIH/AISC	Non-AIH-LD	p
		n (%)	n (%)	
ANA Pattern	fine speckled	30 (39.0 %)	69 (70.4 %)	.000
	homogenous	39 (50.7 %)	22 (22.5 %)	.001
	fine speckled/homogenous	6 (7.8 %)	3 (3.1 %)	.165
	nucleolar	1 (1.3 %)	4 (4.1 %)	.272
	centromer	1 (1.3 %)	0 (0.0 %)	.259

ANA: antinuclear antibodies; AIH: autoimmune hepatitis; AISC: autoimmune sclerosing cholangitis; non-AIH-LD: non-AIH, non-autoimmune sclerosing cholangitis liver disease. Mann-Whitney U test.

Table S3: Test performance of IFT-based autoantibody detection in treatment-naive juvenile AIH vs. non-AIH-LD

ANA IFT	Titer	sensitivity	specificity	PPV	NPV	accuracy
Any tissue positivity	1:20	89,9%	26,7%	41,3%	82,1%	49,7%
	1:40	89,9%	27,5%	41,6%	82,5%	50,3%
	1:80	87,0%	38,3%	44,8%	83,6%	56,1%
	1:160	73,9%	65,8%	55,4%	81,4%	68,8%
	**1:320**	**59,4%**	**85,0%**	**69,5%**	**78,5%**	**75,7%**
	1:640	34,8%	94,2%	77,4%	71,5%	72,5%
HEp-2 cell positivity	1:20	95,7%	18,3%	40,2%	88,0%	46,6%
	1:40	95,7%	20,0%	40,7%	88,9%	47,6%
	1:80	92,8%	30,0%	43,2%	87,8%	52,9%
	1:160	76,8%	60,8%	53,0%	82,0%	66,7%
	**1:320**	**58,0%**	**85,0%**	**69,0%**	**77,9%**	**75,1%**
	1:640	34,8%	93,3%	75,0%	71,3%	72,0%
**SMA IFT**						
any SMA	1:20	92,8%	15,8%	38,8%	79,2%	43,9%
	1:40	91,3%	18,5%	38,9%	77,8%	44,4%
	1:80	78,3%	29,2%	38,9%	70,0%	47,1%
	1:160	65,2%	74,2%	59,2%	78,8%	70,9%
	**1:320**	**50,7%**	**95,0%**	**85,4%**	**77,0%**	**78,8%**
	1:640	34,8%	99,2%	96,0%	72,6%	75,7%
V	1:20	11,6%	75,0%	21,1%	59,6%	51,9%
	1:40	11,6%	75,0%	21,1%	59,6%	51,9%
	1:80	8,7%	79,2%	19,4%	60,1%	53,4%
	1:160	4,4%	92,5%	25,0%	62,7%	60,3%
	**1:320**	**1,5%**	**98,3%**	**33,3%**	**63,4%**	**63,0%**
	1:640	0,0%	1000,0%		63,5%	63,5%
VG	1:20	50,7%	70,0%	49,3%	71,2%	63,0%
	1:40	49,3%	71,7%	50,0%	71,1%	63,5%
	1:80	44,9%	76,7%	52,5%	70,8%	65,1%
	1:160	43,5%	93,3%	79,0%	74,2%	75,1%
	**1:320**	**37,7%**	**99,2%**	**96,3%**	**73,5%**	**76,7%**
	1:640	27,5%	100,0%	100,0%	70,6%	73,5%
VGT	**1:20**	**13,00%**	**100,00%**	**100,00%**	**66,70%**	**68,30%**
	1:40	13,00%	100,00%	100,00%	66,70%	68,30%
	1:80	13,00%	100,00%	100,00%	66,70%	68,30%
	1:160	13,00%	100,00%	100,00%	66,70%	68,30%
	1:320	11,60%	100,00%	100,00%	66,30%	67,70%
	1:640	11,60%	100,00%	100,00%	66,30%	67,70%

ANA: antinuclear antibodies. SMA: anti-smooth muscle antibodies. PPV: positive predictive value. NPV: negative predictive value. V: staining of vessels. VG: staining of vessels and glomeruli. VGT: staining of vessels, glomeruli and tubuli. IFT: immunofluorescence testing.

Table S4: Test performance of ELISA-based autoantibody in treatment-naive juvenile AIH vs. non-AIH-LD

recommended cut offs	ANA				F-Actin		pIgG
	Euroimmun	Bio-Rad	Inova				
Cut-off	≥1.0	≥1.0	>20(-60)	>60	>20 (-30)	>30	1.27 nAU
Sensitivity	24,6%	60,9%	62,3%	20,3%	73,9%	60,9%	78,3%
Specificity	90,8%	80,0%	69,2%	94,2%	80,0%	94,2%	86,7%
PPV	60,7%	63,6%	53,8%	66,7%	68,0%	85,7%	77,1%
NPV	67,7%	78,1%	76,2%	67,3%	84,2%	80,7%	87,4%
Accuracy	66,7%	73,0%	66,7%	67,2%	77,8%	82,0%	83,6%

ANA: antinuclear antibodies. PPV: positive predictive value. NPV: negative predictive value. pIgG: polyreactive immunoglobulin G.

Table S5: Agreement between different assays

		kappa
IFT ANA rodent tissue ≥1/20	IFT ANA HEp-2 ≥1/20	0.71
	pIgG	0.04
	ANA Inova	0.12
	ANA Bio-Rad	0.12
	ANA Euroimmun	0.03
IFT ANA rodent tissue ≥1/320	IFT ANA HEp-2 ≥1/320	0.81
	pIgG	0.32
	ANA Inova	0.31
	ANA Bio-Rad	0.40
	ANA Euroimmun	0.19
IFT ANA HEp-2 ≥1/20	pIgG	0.01
	ANA Inova	0.08
	ANA Bio-Rad	0.07
	ANA Euroimmun	0.02
IFT ANA HEp-2 ≥1/320	pIgG	0.30
	ANA Inova	0.36
	ANA Bio-Rad	0.41
	ANA Euroimmun	0.20
IFT any SMA ≥1/20	F-Actin	0.02
IFT any SMA ≥1/320	F-Actin	0.39
pIgG	F-Actin	0.45

Agreement is assessed by Cohen's Kappa ("Kappa"). Titer is provided as cut-off value for IFT-based assays.

pIgG: polyreactive immunoglobulin G; IFT: immunofluorescence testing; ANA: antinuclear antibodies; SMA: anti-smooth muscle antibodies: HEp-2: human epithelioma 2.

Table S6: Sensitivity and Specificity of ELISA with regard to storage duration

pIgG: polyreactive immunoglobulin G; ANA: antinuclear antibodies. Chi² test.

Table S7: Correlation of IgG with different autoantibody tests

Pearson correlation coefficient is provided. ANA: antinuclear antibodies. SMA: anti-smooth muscle antibodies: HEp-2: human epithelioma 2. pIgG: polyreactive immunoglobulin G. IgG: immunoglobulin G.
